# Contributors to reduced life expectancy among Native Americans in the Four Corners States

**DOI:** 10.1371/journal.pone.0256307

**Published:** 2021-08-17

**Authors:** Olusola A. Omisakin, Hyojun Park, Max T. Roberts, Eric N. Reither

**Affiliations:** Department of Sociology and Anthropology, Utah State University, Logan, Utah, United States of America; Montana State University, UNITED STATES

## Abstract

To assess trends in life expectancy and the contribution of specific causes of death to Native American-White longevity gaps in the Four Corners states, we used death records from the National Center for Health Statistics and population estimates from the U.S. Census Bureau from 1999–2017 to generate period life tables and decompose racial gaps in life expectancy. Native American-White life expectancy gaps narrowed between 2001 and 2012 but widened thereafter, reaching 4.92 years among males and 2.06 years among females in 2015. The life expectancy disadvantage among Native American males was primarily attributable to motor vehicle accidents (0.96 years), liver disease (1.22 years), and diabetes (0.78 years). These causes of deaths were also primary contributors to the gap among females, forming three successive waves of mortality that occurred in young adulthood, midlife, and late adulthood, respectively, among Native American males and females. Interventions to reduce motor vehicle accidents in early adulthood, alcohol-related mortality in midlife, and diabetes complications at older ages could reduce Native American-White longevity disparities in the Four Corners states.

## Introduction

As of January 7, 2021, Navajo Nation reported a total of 24,521 cases of COVID-19 and 844 deaths attributable to the disease [1]. This translates into 49 COVID-19 deaths per 10,000 residents, far exceeding the overall U.S. rate of 11 per 10,000 [2]. Disconcertingly high rates of COVID-19 mortality in Navajo Nation highlight large health disparities faced by this population. Although COVID-19 is the most recent and a particularly urgent expression of these disadvantages, other causes of morbidity and mortality that long predate the SARS-CoV-2 virus have also prematurely shortened lives in Navajo Nation and other nearby populations of Native peoples. In this study, our overarching aim is to disentangle these various causes of death, showing how they contribute to longevity disparities in this understudied population of Native Americans.

With more than 170,000 residents spread across 27,450 square miles in Arizona, New Mexico, and Utah, Navajo Nation is by far the largest and most populous Native American reservation in the United States [3, 4]. It is also directly adjacent to the Ute and Southern Ute reservations in Colorado. Other reservations in these states, often referred to as the Four Corners states (hereafter, FCS) due to a shared border at the quadripoint of 37° north latitude and 109° 03’ west longitude, include the Hopi in Arizona, the Uinta and Ouray in Utah, the Santa Clara Pueblo in New Mexico, and many others [5]. Relative to non-Hispanic Whites and other racial/ethnic groups in the FCS, Native Americans have consistently been disadvantaged with respect to conditions such as diabetes and unintentional injuries, as well as health-risk behaviors like cigarette smoking and chronic drinking [6–9].

However, there is limited understanding of causes that perpetuate longevity disparities for Native Americans who reside in this geographical region. Prior studies have identified unintentional injuries [10], liver disease [10–12], cancer [10–12], and diabetes [10, 11, 13] as contributors to mortality disparities between Native American and White populations in the U.S. Higher mortality among Native Americans has been attributed to adverse socioeconomic conditions [6], behavioral factors including smoking [6], drinking [14, 15], and obesity [9, 16], healthcare disadvantage [6], limited federal funding [17, 18], and rural isolation [10]. While these studies provide useful clues, no study to date has examined the relative contributions of various causes of death to the gap in life expectancy between Native Americans and Whites in the FCS.

Considering the concentration of Native American reservations in the FCS and stark health disparities recently highlighted by COVID-19 in Navajo Nation, it is important to understand what causes of death are primarily responsible for longevity disparities in this region. Therefore, our study has three main objectives: first, we will examine recent trends in longevity disparities between Native Americans and non-Hispanic Whites in the FCS; second, we will build demographic models to assess how various causes of death have contributed to these longevity disparities over time; third, we will measure how the contribution from each cause of death varies by age, allowing us to pinpoint stages in the life course where each cause of death makes the largest contribution to longevity disparities. Knowledge generated through this study will help public health stakeholders develop targeted programs to address mortality disparities among Native Americans in the FCS.

## Materials and methods

### Data sources

We utilized 1999–2017 restricted-use mortality data from the U.S. National Center for Health Statistics (NCHS) [19]. The NCHS compiles data on the health status of each state’s population in the U.S., including records of death. To estimate mortality disparities among Native Americans in the FCS, we used underlying cause-of-death records for non-Hispanic Whites and non-Hispanic Native Americans in Arizona, Colorado, New Mexico, and Utah. We also used bridged-race population estimates from the U.S. Census Bureau from 1999 to 2017 [20].

### Measures

We used measures of sex, age, race/ethnicity, and cause of death in our study. We grouped age at death into 19 distinct categories (less than 1 year, 1–4 years, five-year age groups from 5–9 to 80–84, and 85 and above) for males and females. We restricted our focus to non-Hispanic individuals belonging to either “American Indian or Alaska Native” or “White” populations in the FCS, as previous studies have shown misclassification bias among Hispanic Native Americans (i.e., population data for Hispanic Native Americans tend to be overestimated, resulting in underestimation of death rates) [12]. In addition, Hispanics have socioeconomic and health characteristics that are distinct from non-Hispanic populations [21, 22]; thus, our analysis is limited to non-Hispanics in the FCS.

Using the International Classification of Disease (ICD) 10^th^ revision, we compiled two different cause-of-death lists. First, we examined the 10 leading causes of death in the United States, namely heart disease, cancer, unintentional injury, chronic lower respiratory disease, cerebrovascular disease, Alzheimer’s disease, diabetes, influenza and pneumonia, nephritis, and suicide [23]. Based on epidemiological evidence from existing literature [10–13] and our preliminary analyses, the second list was comprised of 11 causes that are especially relevant to Native Americans in the FCS. We included only causes of death that met a contribution threshold of 0.2-years to the Native American-White life expectancy gap during any single period for either males or females. These include accidental poisoning, diabetes, influenza and pneumonia, mental disorder from alcohol use, motor vehicle accidents, nephritis, other unintentional injuries (e.g., falls), homicide, other infectious diseases (e.g., HIV), liver disease, and remaining U.S. leading causes. In this second set of ICD measures, we disaggregated the unintentional injury category to separate accidental poisoning from motor vehicle deaths. For each set of causes, we included a residual category to capture all other causes of death.

### Analysis

The first step in our analysis was to calculate a series of period life tables for Native Americans and Whites, spanning the period of observation in our study. Using death records from the NCHS and bridged-race population estimates from the U.S. Census Bureau, we calculated mortality rates for specific age, race, and sex categories. Next, we used these mortality rates to estimate life expectancies at birth for Native American and White males and females, following a standard procedure for creating abridged life tables [24]. We accounted for random annual fluctuations in period mortality by combining data into five-year aggregates of deaths and population estimates (e.g., 2001 represents 1999 to 2003), similar to approaches used in previous research [25].

The second step in our analysis was to decompose life expectancy gaps between Native Americans and Whites into various causes of death. We decomposed the life expectancy gap at three different time points (2001, 2008, and 2015) into portions attributable to specific causes of death, as well as a residual category for other causes of death, using Arriaga’s approach [24, 26]. Next, we calculated the number of years that each cause contributed to the Native American-White life expectancy gap at specific ages, revealing life stages where each cause of death contributed most to longevity disparities. Period life tables and decomposition analyses were calculated using SAS 9.4 and code provided by Auger et al. [26].

## Results

**Fig 1:**
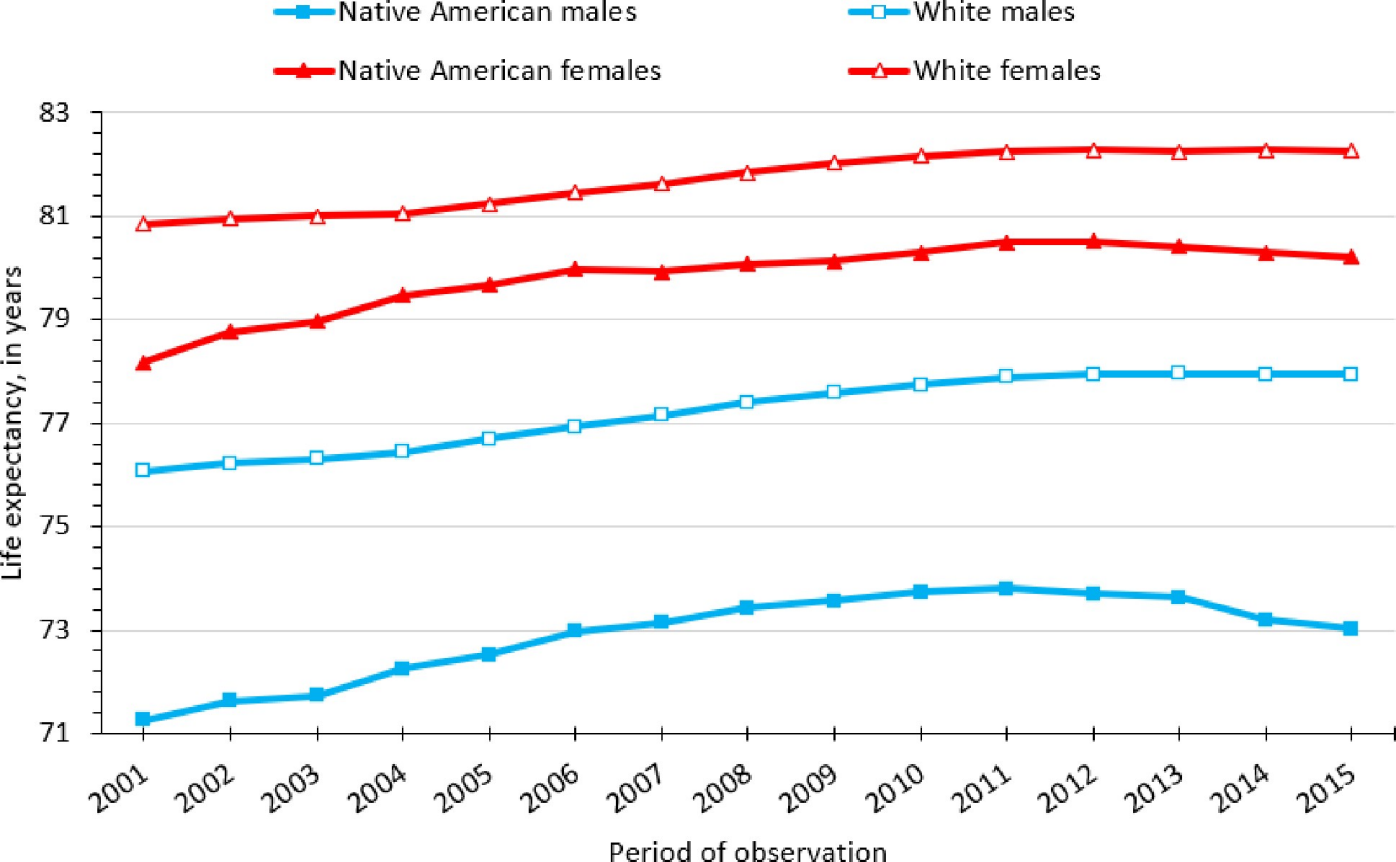
Life expectancy among Native American and White in the Four Corners States, 2001–2015. Each period represents a 5-year aggregation of data.

Over our entire period of observation (2001–2015), Whites could expect to live 3.0 more years than Native Americans in the FCS. This overall difference in life expectancy at birth varied over time and by sex, as depicted in Fig 1. Despite an overall increase in life expectancy among all race-sex groups, we observed persistent disadvantages among Native Americans. Among males, the Native American-White life expectancy gap narrowed from 4.81 years in 2001 (i.e., 76.06 vs. 71.25 for Whites and Native Americans, respectively) to 3.98 years in 2008, before widening to 4.92 years in 2015. We found smaller longevity gaps among females, although disparity patterns were similar to males. The Native American-White life expectancy gap among females declined from 2.67 years in 2001 (i.e., 80.85 vs. 78.17 for Whites and Native Americans, respectively) to 1.76 years in 2008, before increasing to 2.06 years in 2015.

Table 1 presents contributions of the 10 leading U.S. causes of death to Native American-White life expectancy gaps in 2001, 2008, and 2015. For each cause of death in each time period, contributions were quantified as the years gained or lost among Native Americans compared to Whites. Our analyses revealed that unintentional injuries, diabetes, and influenza/pneumonia were consistently large contributors to the persistent longevity gap. In 2015, for example, these three causes of death contributed 2.81 years (i.e., 2.81 = 1.81+0.78+0.22) to the Native American-White gap among males and 1.68 years to the gap among females. Unintentional injuries were the largest contributor to Native American-White longevity gaps among males (2.19, 1.64, and 1.81 years in 2001, 2008, and 2015 respectively). Among females, diabetes was the largest contributor to the Native American-White longevity gap, followed closely by unintentional injuries. Several leading causes of death in the U.S. consistently favored Native Americans among both males and females, including heart disease, cancer, chronic lower respiratory disease, and Alzheimer’s disease. Neither suicide nor cerebrovascular disease made noteworthy contributions to racial disparities in longevity. In each period of observation, there were large residuals (i.e., portions of Native American-White longevity gaps not explained by the 10 leading U.S. causes of death), as shown via the “all other causes” category.

**Table 1:** Contribution of ten leading U.S. causes of death to Native American-White life expectancy gaps in the Four Corners States.

Cause of Death	Males			Females		
	2001	2008	2015	2001	2008	2015
White life expectancy	76.06	77.40	77.94	80.85	81.84	82.26
Native American life expectancy	71.25	73.43	73.02	78.17	80.08	80.20
**Racial e**_**0**_ **Gap**	**4.81**	**3.98**	**4.92**	**2.67**	**1.76**	**2.06**
Heart disease	-0.23	-0.28	-0.08	-0.39	-0.49	-0.56
Cancer	-0.72	-0.67	-0.53	-0.60	-0.58	-0.50
Unintentional injuries	2.19	1.64	1.81	0.96	0.53	0.65
Chronic lower respiratory disease	-0.38	-0.38	-0.36	-0.43	-0.52	-0.50
Cerebrovascular disease	0.00	0.02	0.03	-0.11	-0.14	-0.08
Alzheimer’s disease	-0.10	-0.16	-0.17	-0.24	-0.36	-0.43
Diabetes	0.64	0.64	0.78	0.97	0.80	0.78
Influenza and pneumonia	0.34	0.33	0.22	0.35	0.37	0.25
Nephritis	0.16	0.15	0.11	0.32	0.30	0.16
Suicide	0.15	0.02	-0.07	-0.04	-0.04	0.00
All other causes	2.77	2.68	3.18	1.87	1.90	2.29

Note: Each period represents a 5-year aggregation of data.

To help explain large residuals, we evaluated 11 causes of death that are particularly relevant to Native American communities in the FCS (Table 2). Despite some fluctuations by year and sex, the three leading contributors to the Native American-White longevity gap were consistently liver disease, motor vehicle accidents, and diabetes. Combined, these three causes of death accounted for more than 60% of the racial longevity gap observed among males and more than 90% of the disparity observed among females in each period of observation. Among males, other unintentional injuries, homicide, and mental disorders from alcohol use individually contributed between 0.39 and 0.59 years to the gap in 2001, 2008, and 2015. Other notable contributors among males included accidental poisoning (0.37 years in 2015), other infectious diseases (0.34 years in 2015), and influenza and pneumonia (0.22 years in 2015). Among females, influenza and pneumonia, other infectious diseases, and nephritis also made notable contributions to the longevity gap, particularly in 2008. Relative to the decomposition model in Table 1, the model in Table 2 has much smaller residuals, as shown by the “all other causes” category.

**Table 2:** Contribution of eleven selected causes of death to Native American-White life expectancy gaps in the Four Corners States.

Cause of Death	Males			Females		
	2001	2008	2015	2001	2008	2015
White life expectancy	76.06	77.40	77.94	80.85	81.84	82.26
Native American life expectancy	71.25	73.43	73.02	78.17	80.08	80.20
**Racial e**_**0**_ **Gap**	**4.81**	**3.98**	**4.92**	**2.67**	**1.76**	**2.06**
Unintentional injuries						
Accidental poisoning	0.05	0.19	0.37	0.00	-0.05	0.00
Motor vehicle accidents	1.56	0.91	0.96	0.81	0.54	0.55
Other unintentional injuries	0.59	0.54	0.48	0.15	0.05	0.09
Diabetes	0.64	0.64	0.78	0.97	0.80	0.78
Influenza and pneumonia	0.34	0.33	0.22	0.35	0.37	0.25
Nephritis	0.16	0.15	0.11	0.32	0.30	0.16
Remaining U.S. leading causes^†^	-1.30	-1.45	-1.18	-1.81	-2.13	-2.06
Liver disease	0.99	0.88	1.22	0.80	0.99	1.27
Other infectious diseases	0.23	0.38	0.34	0.32	0.38	0.30
Mental disorders from alcohol use	0.49	0.40	0.41	0.14	0.11	0.15
Homicide	0.47	0.47	0.39	0.16	0.10	0.09
All other causes	0.59	0.55	0.82	0.45	0.31	0.47

Note: Each period represents a 5-year aggregation of data.

^†^ Includes heart disease, cancer, chronic lower respiratory disease, cerebrovascular disease, Alzheimer’s disease, and suicide.

**Fig 2:**
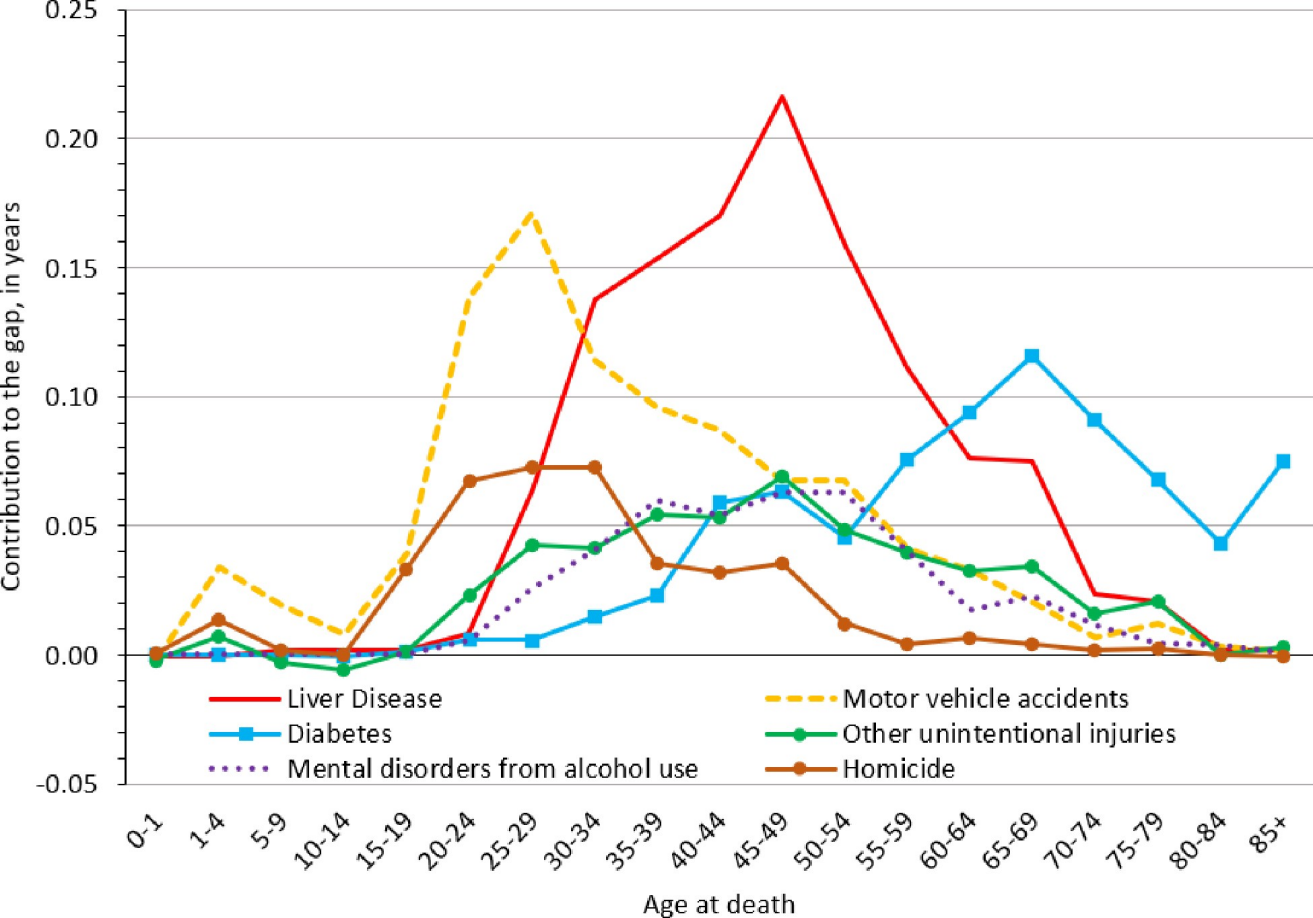
Native American-White life expectancy gap in the Four Corners States among males, 2015. The life expectancy gap was decomposed into various causes of death at specific ages. Estimates for 2015 represent a 5-year aggregation of data (i.e., 2013–2017).

In addition to overall contributions for each cause of death shown in Table 2, we determined how these contributions varied across the life course for the six most influential causes of death among males and females. Because we observed generally stable cause-of-death and age patterns across different periods, we present results for the latest year of observation (i.e., 2015), which is also likely to be most relevant for interventions and policies that could be informed by our findings. As Fig 2 shows, motor vehicle accidents and homicide made large contributions to the Native American-White life expectancy gap among young adult males. The contribution from motor vehicle accidents was most pronounced at age 25–29 (0.17 years); homicide’s peak contribution occurred at age 30–34 (0.07 years). As motor vehicle accidents and homicide waned among males in their late 30s and 40s, liver disease overtook these conditions as the largest contributor to longevity gaps until males reached their late 50s. Contributions from liver disease were especially large at ages 40–44 (0.16 years), 45–49 (0.22 years), and 50–54 (0.18 years). During this period of the life course, the “boom” in liver disease among Native American males was echoed by mental disorders from alcohol use and other unintentional injuries, which both followed broadly similar age patterns. Around retirement age, diabetes became the largest contributor to the longevity gap among males, and it retained this position for the remainder of the life course. Diabetes was a particularly notable contributor at ages 60–64 (0.09 years), 65–69 (0.12 years), and 70–74 (0.08 years).

**Fig 3:**
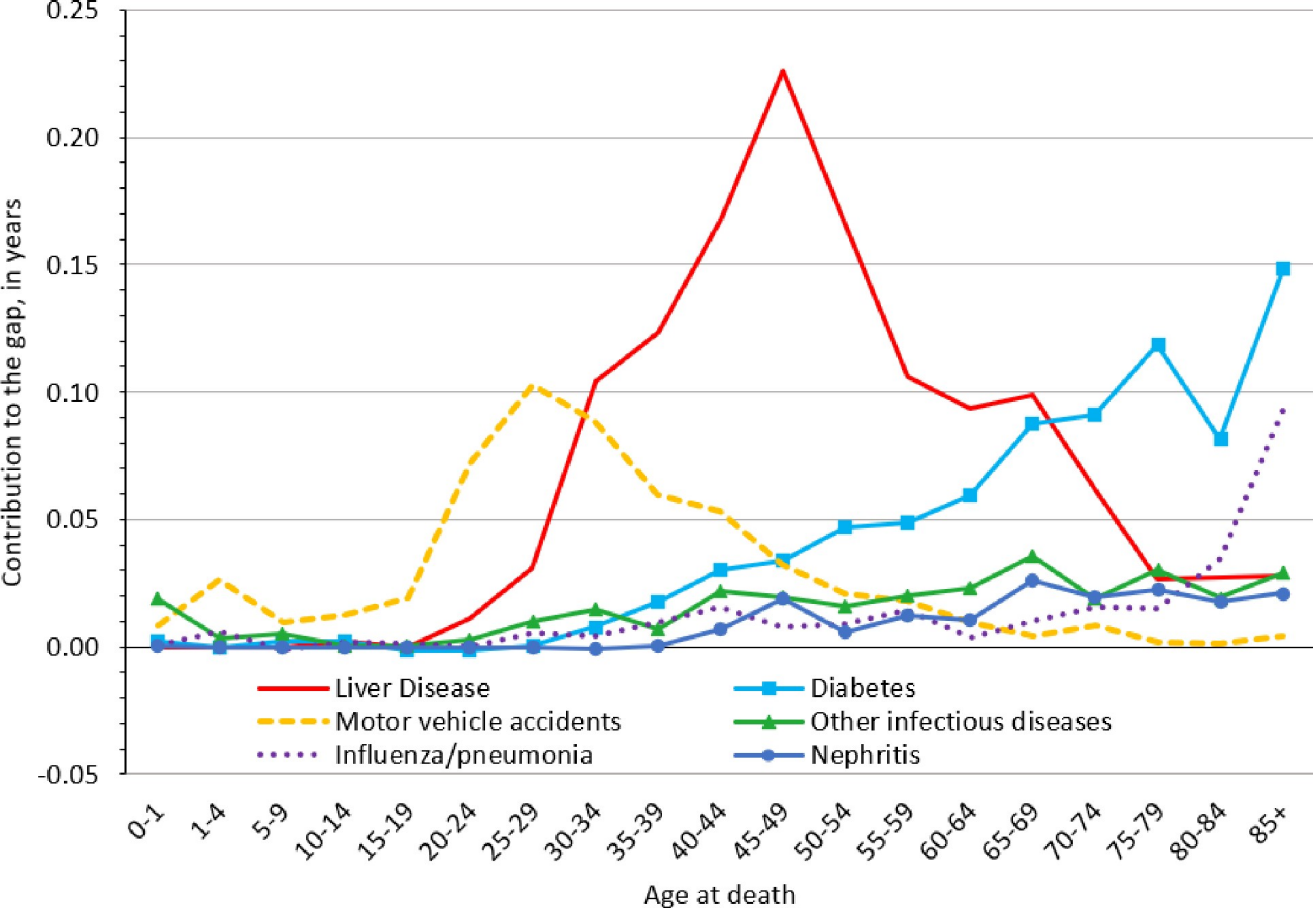
Native American-White life expectancy gap in the Four Corners States among females, 2015. The life expectancy gap was decomposed into various causes of death at specific ages. Estimates for 2015 represent a 5-year aggregation of data (i.e., 2013–2017).

In Fig 3, we present age patterns for the six largest contributors to the Native American-White longevity gap among females in 2015. In early life, the main contributor to the longevity gap was motor vehicle accidents, particularly between ages 20–34. Similar to what we observed among males, the peak contribution from motor vehicle accidents occurred at age 25–29 years among females (0.11 years). Also similar to the pattern among males, liver disease surpassed motor vehicle accidents among females in their late 30s and 40s, peaking at age 45–49 years (0.23 years). Liver disease retained its position as the most important contributor to the Native American-White longevity gap among females until age 70, when it was finally overtaken—first by diabetes, and subsequently by influenza and pneumonia. Diabetes contributed most to the gap between ages 70–74 (0.09 years), 75–79 (0.12 years), and 85 and above (0.15 years); influenza and pneumonia contributed most at ages 80–84 (0.05 years) and 85 and above (0.09 years). Other infectious diseases and nephritis were, by comparison, relatively small contributors that became increasingly evident in mid-to-late life.

## Discussion

Consistent with previous studies [10–12], we observed persistent life expectancy disadvantages among Native Americans in recent decades. We extended prior research by examining these disparities in the FCS region of the U.S., which includes Navajo Nation and several other large groups of Native Americans. Our study also extends prior work by identifying specific life stages where each cause of death contributes most to life expectancy gaps between Native Americans and Whites. Through this approach, we found that three major waves of mortality among Native Americans in the FCS accounted for a large proportion of longevity disparities observed in this region.

The first wave of mortality was produced by motor vehicle accidents among Native American males and females in early adulthood. During this stage of life, homicide also made notable contributions to the longevity gap between Native American and White males. Both contributors peaked in the mid 20s but remained important causes of mortality disparities for Native Americans in their 30s and 40s. Our findings are consistent with prior research in the FCS. For instance, a 2013 report on health disparities in the state of New Mexico found that (a) motor vehicle death rates among Native Americans were three times higher than Whites, and (b) homicide among Native Americans caused 14.7 deaths per 100,000 residents, compared to just 3.6 per 100,000 among Whites [9]. In addition to research in the FCS, our findings support prior studies of Native Americans in urban areas and other regions of the U.S., which have reported high rates of motor vehicle-related mortality among young adults [10]. Factors contributing to the first wave of Native American mortality in the FCS could include lower rates of seat belt use and child safety seat use among Native Americans, which increases the lethality of car accidents. In addition, alcohol consumption and substance use among adolescent and young adult Native Americans in the FCS may contribute to accidents and interpersonal conflicts that result in fatalities [6, 10].

The second wave of mortality among Native Americans in the FCS was propelled primarily by liver disease. For both male and females, this wave of mortality ascended rapidly in the late 20s, crested in the late 40s, and diminished steadily with age thereafter. During this stage of life, mental disorders from alcohol use and other unintentional injuries also made important contributions to the Native American-White longevity gap among males. We note that drug overdoses make up a portion of accidental poisoning in our analyses, altogether contributing more than a third of a year to the Native American-White longevity gap among males in 2015. The disconcertingly large impact of liver disease and other alcohol-related conditions on premature mortality among Native Americans in the FCS may be related to high rates of alcohol consumption. This attribution is challenged by a recent study of national survey data, which found comparable rates of heavy and binge drinking among Native Americans and non-Hispanic Whites [27]. However, these national data are inconsistent with some evidence within the FCS. For instance, the Utah Department of Health has reported that chronic drinking among Native Americans is nearly three times higher than the general population in the state [6]. Similarly, in New Mexico between 2013–17, alcohol-related chronic diseases caused 102 deaths per 100,000 Native American residents, compared to 21 deaths per 100,000 non-Hispanic White residents [14]. Although alcohol consumption likely affects mortality disparities from liver disease in the FCS, other causes of liver disease such as hepatitis and obesity also disproportionately affect Native Americans [28]. Future research should attempt to isolate the relative impact of these contributors to liver disease in the FCS.

The third wave of mortality was driven principally by diabetes in mid-to-late life. To a lesser extent, influenza, pneumonia, and other infectious diseases also contributed to this final wave of mortality. The disproportionate mortality burden attributable to diabetes may be related to higher rates of physical inactivity and obesity among Native Americans in the FCS. To illustrate, the 2017 Behavioral Risk Factor Surveillance System (BRFSS) in Arizona indicated that 31.1% of Native Americans reported no leisure-time physical activity, compared to 23.0% of non-Hispanic Whites [7]. Similarly, a study in Utah found that 29.0% of Native Americans reported no leisure-time physical activity, compared to 18.3% of the general population [6]. Data from the 2017 BRFSS also show that consumption of sugary beverages was somewhat higher among Native Americans (33.0%) in Arizona than among non-Hispanic Whites (26.8%) in the state [7]. Obesity prevalence among Native Americans and Whites was 39.2% and 22.1%, respectively, in New Mexico in 2011–12 [9], reflecting generally higher obesity rates among Native Americans who reside within the FCS.

### Public health implications

Reducing life expectancy disparities in the FCS will require programs and policies capable of addressing the complex set of risk factors that contribute to three major waves of mortality across the life course of Native Americans. For example, programs that address alcohol and other substance use in early through mid-adulthood could reduce fatal accidents as well as liver disease mortality, diminishing contribtutions from these causes of death to the first two waves of mortality. Similarly, policies that promote equity in access to healthy foods and exercise amenities such as parks and walking trails in the FCS could reduce the third wave of mortality among Native Americans, caused primarily by diabetes. While directly addressing the proximate determinants of accidents, liver disease, and diabetes is important, it is also necessary to consider underlying “fundamental causes” of mortality disparities—including unequal access to education, healthcare, basic income, and other essential resources [29].

Future policies should also strive to reduce the burden of influenza, pneumonia, and other infectious disease mortality among Native Americans in the FCS. Greater susceptibility to these and novel infectious diseases (e.g., COVID-19) may be attributable to factors such as the tendency to live in multigenerational households, poor indoor air quality, inadequate household plumbing (which can make frequent handwashing difficult), and inadequate access to grocery stores where healthy food is accessible [17, 30]. Achieving racial equity in infectious disease burden will require policies that address these issues as well as immunization against infectious diseases, which tends to be much lower among Native Americans than Whites in the FCS [9]. As a new infectious disease, COVID-19 is likely exacerbating the Native American-White longevity gap in the FCS, beyond the information conveyed through our data. Morever, given the severity of the COVID-19 epidemic among Native Americans in the FCS, there is an urgent need to promote COVID-19 vaccination and ensure that adequate vaccine supplies are distributed throughout Native communities.

### Strengths and limitations

One key strength of our study is the analysis of restricted-use NCHS data, eliminating the need to account for missing data in public records (e.g., via imputation techniques). Another important strength is the evaluation of numerous causes of death over the life course of Native Americans, facilitating our discovery that three major waves of mortality accounted for most of the racial longevity gap in the FCS. Although our analyses explain more than 75% of the life expectancy gap among males and females in 2015, we could not fully explain these disparities; future research may want to consider additional contributors. Another limitation of our study is that mortality data are not linked with socioeconomic measures. While we acknowledge the importance of social and economic disadvantages faced by Native Amiericans in the FCS, we are unable to quantify how much these factors contribute to racial disparities in longevity. Future research could evaluate how social and economic disadvantages contribute to the three waves of mortality identified among Native Americans in this study. Finally, misclassification of Native American identity in both death certificates and population estimates tends to inflate life expectancy for Native Americans [31]; thus, our calculated life expectancy gaps are likely underestimated. As such biases tend to be small among Native Americans living in reservation areas [32], we expect that the impact of misclassification bias is modest and toward the null, making our findings conservative.

## Conclusions

We found large and persistent life expectancy gaps between non-Hispanic Native Americans and non-Hispanic Whites in the FCS. This ongoing inequity presents an urgent challenge to the goals of Healthy People 2030 and other calls for racial justice in health and longevity. Our investigation also revealed that three major waves of mortality among Native Americans in the FCS—motor vehicle accidents in early adulthood, liver disease in midlife, and diabetes at older ages—contributed most to the life expectancy gap in 2015. Reducing Native American-White disparities in longevity will require programs and policies that are (a) culturally appropriate and tailored to the needs of Native Americans in the FCS, and (b) address the determinants of accidents, liver disease, and diabetes, such as substance use and obesity. Future policies should also account for fundamental underlying contributors, such as unequal access to healthcare, education, and basic income.

## References

[1] Navajo Department of Health. Navajo Nation COVID-19 Dashboard. 2021 [cited 21 Mar 2021]. Available: https://www.ndoh.navajo-nsn.gov/COVID-19/Data

[2] JHU CSSE–Center For Systems Science and Engineering at JHU. 2021 [cited 21 Mar 2021]. Available: https://systems.jhu.edu/

[3] Centers for Disease Control and Prevention. Tribal Reservations. 2018 [cited 24 Sep 2020]. Available: https://www.cdc.gov/tribal/tribes-organizations-health/tribes/reservations.html

[4] U.S. General Services Administration. Tribal Nations Maps. In: Data.gov [Internet]. [cited 24 Sep 2020]. Available: https://www.data.gov/climate/tribal-nations/tribal-nations-maps/

[5] The Navajo Epidemiology Center. Navajo Population Profile 2010 U.S. Census. 2013.

[6] The Utah Department of Health. Utah Health Disparities Summary 2009. American Indians Chronic Conditions, Reproductive Health, Injury and Lifestyle Risk. 2009. Available: http://health.utah.gov/disparities/data/ohd/archives/AmericanIndianFactSheet.pdf

[7] Adakai M, Sandoval-Rosario M, Xu F, Aseret-Manygoats T, Allison M, Greenlund KJ, et al. Health Disparities Among American Indians/Alaska Natives—Arizona, 2017. 2018;67: 5. doi: 10.15585/mmwr.mm6747a4 30496159 PMC6276383

[8] Colorado Department of Public Health and Environment. 2015 Health Disparity Fact Sheet American Indians/Alaska Natives in Colorado. 2015. Available: https://www.colorado.gov/pacific/sites/default/files/OHE-CO-American-Indian-Health-Disparities-Fact-Sheet-2015.pdf

[9] New Mexico Department of Health. American Indian Health Equity: A Report on Health Disparities in New Mexico. 2013. Available: https://www.nmhealth.org/publication/view/report/44/

[10] Jacobs-Wingo JL, Espey DK, Groom AV, Phillips LE, Haverkamp DS. Causes and Disparities in Death Rates Among Urban American Indian and Alaska Native Populations, 1999–2009. Am J Public Health. 2016;106: 906–914. doi: 10.2105/AJPH.2015.303033 26890168 PMC4985112

[11] Woolf SH, Chapman DA, Buchanich JM, Bobby KJ, Zimmerman EB, Blackburn SM. Changes in midlife death rates across racial and ethnic groups in the United States: systematic analysis of vital statistics. BMJ. 2018; k3096. doi: 10.1136/bmj.k3096 30111554 PMC6092678

[12] Espey DK, Jim MA, Cobb N, Bartholomew M, Becker T, Haverkamp D, et al. Leading Causes of Death and All-Cause Mortality in American Indians and Alaska Natives. Am J Public Health. 2014;104: S303–S311. doi: 10.2105/AJPH.2013.301798 24754554 PMC4035872

[13] Espey D, Paisano R, Cobb N. Regional patterns and trends in cancer mortality among American Indians and Alaska Natives, 1990–2001. Cancer. 2005;103: 1045–1053. doi: 10.1002/cncr.20876 15685622

[14] NM-IBIS—Complete Health Indicator Report—Alcohol—Alcohol-related Chronic Disease Deaths. [cited 21 Mar 2021]. Available: https://ibis.health.state.nm.us/indicator/complete_profile/AlcoholRelatedDthChronic.html

[15] Delker E, Brown Q, Hasin DS. Alcohol Consumption in Demographic Subpopulations. Alcohol Res Curr Rev. 2016;38: 7–15. 27159807 10.35946/arcr.v38.1.02 PMC4872616

[16] Bullock A, Sheff K, Moore K, Manson S. Obesity and Overweight in American Indian and Alaska Native Children, 2006–2015. Am J Public Health. 2017;107: 1502–1507. doi: 10.2105/AJPH.2017.303904 28727519 PMC5551606

[17] Kakol M, Upson D, Sood A. Susceptibility of Southwestern American Indian Tribes to Coronavirus Disease 2019 (COVID‐19). J Rural Health. 2020 [cited 28 Jul 2020]. doi: 10.1111/jrh.12451 32304251 PMC7264672

[18] U.S. Commission on Civil Rights. Broken Promises: Continuing Federal Funding Shortfall for Native Americans. 2018. Available: https://www.usccr.gov/pubs/2018/12-20-Broken-Promises.pdf

[19] Mortality data: National Center for Health Statistics. Multiple Cause of Death Mortality—all county (micro-data) files, as compiled from data provided by the 57 vital statistics jurisdictions through the Vital Statistics Cooperative Program (2018).

[20] Centers for Disease Control and Prevention. Population estimates: United States Census Bridged-Race Population Estimates. 2018 [cited 29 Jun 2021]. Available: https://wonder.cdc.gov/controller/datarequest/D163

[21] MORALES LS, LARA M, KINGTON RS, VALDEZ RO, ESCARCE JJ. SOCIOECONOMIC, CULTURAL, AND BEHAVIORAL FACTORS AFFECTING HISPANIC HEALTH OUTCOMES. J Health Care Poor Underserved. 2002;13: 477–503. doi: 10.1177/104920802237532 12407964 PMC1781361

[22] Firebaugh G, Warner C, Massoglia M. Fixed Effects, Random Effects, and Hybrid Models for Causal Analysis. In: Morgan SL, editor. Handbook of Causal Analysis for Social Research. Dordrecht: Springer Netherlands; 2013. pp. 113–132. doi: 10.1007/978-94-007-6094-3_7

[23] Heron M. Deaths: Leading Causes for 2017. Natl Vital Stat Rep. 2019;68: 77. 32501203

[24] Preston SH, Heuveline P, Guillot M. Demography: measuring and modeling population processes. Malden, MA: Blackwell Publishers; 2001.

[25] Roberts MT, Reither EN, Lim S. Contributors to Wisconsin’s persistent black-white gap in life expectancy. BMC Public Health. 2019;19: 891. doi: 10.1186/s12889-019-7145-y 31277617 PMC6612087

[26] Auger N, Feuillet P, Martel S, Lo E, Barry AD, Harper S. Mortality inequality in populations with equal life expectancy: Arriaga’s decomposition method in SAS, Stata, and Excel. Ann Epidemiol. 2014;24: 575–580.e1. doi: 10.1016/j.annepidem.2014.05.006 24970490

[27] Cunningham JK, Solomon TA, Muramoto ML. Alcohol use among Native Americans compared to whites: Examining the veracity of the ‘Native American elevated alcohol consumption’ belief. Drug Alcohol Depend. 2016;160: 65–75. doi: 10.1016/j.drugalcdep.2015.12.015 26868862

[28] Suryaprasad A, Byrd KK, Redd JT, Perdue DG, Manos MM, McMahon BJ. Mortality Caused by Chronic Liver Disease Among American Indians and Alaska Natives in the United States, 1999–2009. Am J Public Health. 2014;104: S350–S358. doi: 10.2105/AJPH.2013.301645 24754616 PMC4035868

[29] Phelan JC, Link BG, Tehranifar P. Social Conditions as Fundamental Causes of Health Inequalities: Theory, Evidence, and Policy Implications. J Health Soc Behav. 2010;51: S28–S40. doi: 10.1177/0022146510383498 20943581

[30] Ehlers CL, Gizer IR. Evidence for a Genetic Component for Substance Dependence in Native Americans. Am J Psychiatry. 2013;170: 154–164. doi: 10.1176/appi.ajp.2012.12010113 23377636 PMC3603686

[31] Dankovchik J, Hoopes MJ, Warren-Mears V, Knaster E. Disparities in life expectancy of pacific northwest American Indians and Alaska natives: analysis of linkage-corrected life tables. Public Health Rep Wash DC 1974. 2015;130: 71–80. doi: 10.1177/003335491513000109 25552757 PMC4245288

[32] Jim MA, Arias E, Seneca DS, Hoopes MJ, Jim CC, Johnson NJ, et al. Racial Misclassification of American Indians and Alaska Natives by Indian Health Service Contract Health Service Delivery Area. Am J Public Health. 2014;104: S295–S302. doi: 10.2105/AJPH.2014.301933 24754617 PMC4035863

